# Quantification of Mesophyll Resistance and Apoplastic Ascorbic Acid as an Antioxidant for Tropospheric Ozone in Durum Wheat (*Triticum durum* Desf. cv. Camacho)

**DOI:** 10.1100/tsw.2008.149

**Published:** 2008-12-14

**Authors:** Daniel de la Torre

**Affiliations:** Universidad Politécnica de Madrid, Plant Biology Department, ETSI Agrónomos, 28040 Madrid, Spain

**Keywords:** apoplastic ascorbic acid, detoxification, mesophyll resistance, ozone, SODA

## Abstract

The daily variations in cellular and apoplastic ascorbic acid and dehydroascorbic acid levels in a Mediterranean durum wheat cultivar (*Triticum durum* Desf. cv. Camacho) were analyzed in order to relate them to ambient ozone exposure and to subsequent stomatally absorbed ozone fluxes. The aim of this study is to prove the effectiveness and accuracy of a computer model (SODA) to calculate the mesophyll resistance (r_m_) to ozone uptake, the percentage of ozone detoxification by apoplastic ascorbic acid, and the ozone flux to the plasmalemma (F_m_) in a Mediterranean durum wheat cultivar. These calculated factors were related to apoplastic ascorbic acid levels and to ambient ozone concentrations. These relationships were obtained with a view to explaining the detoxification of ozone by apoplastic ascorbic acid. Ozone detoxifications of up to 52% were found at midday, when maximum ozone concentrations and maximum apoplastic ascorbic acid are seen. Mesophyll resistance was minimum at this time, and ozone flux to the plasmalemma was reduced because of the reaction of ozone with apoplastic ascorbic acid.

### INTRODUCTION

Scientific evidence has revealed that tropospheric ozone is a highly phytotoxic pollutant and that it is able to cause a decrease in cultivar yields, alter the composition and diversity of some ecosystems, and contribute to a decrease in forest vitality[4,6,9,14,15].

Current ozone toxicity thresholds are based on exposure to the pollutant and the biological response parameters[40]. Nevertheless, the response of the vegetation to atmospheric pollution is strongly dependent on the amount of pollutant that enters the leaf and the resistance of the plants themselves[7,12,13,16,32,38,41]. By means of resistance models, currently intense efforts are being made to characterize ozone deposition in order to establish new ozone threshold values based on ozone uptake fluxes[8,10,41].

The main entry of ozone into plants is through the stomata[19,36] and, hence, stomatal resistance ($r_s$) is one of the most important factors for ozone absorption and, in turn, for injury to plants. Once ozone has entered the substomatal cavity, it contacts the extracellular environment, undergoing dilution and reaction processes that constitute the last line of defense to the entrance of ozone and its reaction products into the inside of the cell. These processes determine mesophyll resistance ($r_m$).

The ozone in the extracellular environment reacts with water and with cellular components to generate free radicals and active forms of oxygen[27,31]. Once formed, these react with multiple organic compounds and oxidize them, depending on their sensitivity and accessibility, resulting in the first injuries that occur at cellular scale[17,18]. In the extracellular (apoplastic) space, some compounds perform a protective-antioxidant function, in particular ascorbic acid[2,24,33]. Ascorbic acid (C vitamin) is a normal constituent of the cells of plants and animals whose main function is to act as an antioxidant. Several authors have described the presence of ascorbic acid in the apoplast, representing about 1% of total cellular ascorbate[2,25,26]. Apoplastic ascorbic acid (ascorbate) is oxidized to dehydroascorbic acid (dehydroascorbate), but the enzymes required for its retransformation into ascorbate are not present in the apoplast, and ascorbate must be transported into the interior of cells[2,21,24]. Cytosolic dehydroascorbate reductase (DHAR) is a key component of this ascorbate recycling system, as reported for *Arabidopsis thaliana* (L.) Heynh[43].

The inverse relationship that has been found in many species between cellular ascorbate and the ozone sensitivity of plants highlights the importance of ascorbate as one of the first lines of defense against ozone[2,6,43,45]. In particular, the small proportion of ascorbate present in the apoplast could be sufficiently large to provide a significant defense against ozone[25,26,28,39]. Additionally, the oxidative stress caused by ozone seems to stimulate the conversion of dehydroascorbate to ascorbate[23,43] and the transport of ascorbate from the symplasm to the apoplast[30,35,43]. Owing to the importance of ascorbate as an antioxidant agent against ozone, it could be considered one of the main elements that would determine mesophyll resistance to ozone deposition in plants.

Some authors have considered that mesophyll resistance is negligible in comparison with stomatal resistance[11]. Nevertheless, the importance of mesophyll resistance relies on the fact that it is the cell's last line of defense before a pollutant is able to gain entry to the inside of the cell. In view of the scarce information available about the reactions that occur in the apoplast, the quantification of mesophyll resistance is hugely complicated. One of the few approaches developed for such purposes is the SODA model (Simulated Ozone Detoxification in the Apoplast), developed by Plöchl and collaborators[34], which was used in this study to estimate mesophyll resistance and the detoxifying role of ascorbate in the apoplast.

### MATERIALS AND METHODS

The experiment was conducted at "El Encín" (40º 31' N 3º 22' W, Alcalá de Henares, Madrid, Spain), an agricultural area belonging to IMIDRA (Institute of Agricultural Research of Madrid). Two plots of durum wheat (*Triticum durum* Desf. cv. Camacho), 5 × 5 $m^2$, were employed and the usual Mediterranean agricultural practices were followed. The Camacho commercial variety was chosen because, based on a preliminary study (data not shown), it is considered as being quite sensitive to ozone. The study was performed during 2 weeks (May 12–23) when the plants were at the phenological stage of anthesis. During this time, the daily profiles of ambient ozone concentrations, the gas exchange rate (stomatal conductance) of the plants, the apoplastic and whole leaf ascorbic acid contents, apoplastic pH, and certain anatomical parameters were analyzed. All measurements were taken from central flag leaf fragments. Quantification of mesophyll resistance ($r_m$), the percentage of ozone reacting with apoplastic ascorbate and the ozone flux to the plasmalemma, was accomplished using the SODA model[34].

### Ambient Ozone Concentrations

Ambient ozone concentrations were recorded using an ultraviolet-photometry ozone analyzer (Dasibi Env. Corp. Model 1003-RS; Glendale, CA). The analyzer was located in a meteorological tower in a wheat field. The air intake was 2.5 m above the ground and the ozone data were registered continuously and automatically every 10 min and stored on a data logger (Geonica Meteodata 1256C; Madrid, Spain).

### Stomatal Conductance ($g_s$) Measurements

Stomatal conductance to water vapor ($g_s$, mol $H_2O$ $m^{-2}$ $s^{-1}$) measurements were made with a portable Li-Cor 6400 gas exchange analyzer (Li-Cor; Lincoln, NE) over two 24-h cycles (one for each week of the measurements), measuring randomly in flag leaves and avoiding the border effect. Between four to 15 samples per hour were taken, depending on the time availability.

### Biochemical Analyses

A total of 24 samples (i.e., corresponding to 1 day) were taken over 1 week, followed by the same protocol over the next week (replicate samples) for the determination of apoplastic ascorbate, whole leaf ascorbate, and apoplastic pH. Six to eight samples per hour were taken for ascorbate determinations. For apoplastic pH determinations, eight to 10 samples per hour were taken to obtain a single hourly value.

The efficiency of the method used to obtain apoplastic fluid was analyzed based on the presence of glucose-6-phosphate-dehydrogenase (G6PDH). This enzyme is only found inside cells and, hence, its activity in apoplastic fluid would indicate a contamination of the latter by cytoplasmic fluid. G6PDH activity was analyzed at four moments of the daily cycle: morning, midday, evening, and night.

### Preparation of Cellular Extracts

Extraction was accomplished following the protocol of Takahama and Oniki[37]. Central fragments of flag leaf (2–3 cm long) were used for ascorbate determinations; for this, they were weighed (Cobos precision balance; Madrid, Spain) and homogenized in 1 mL of ice-cold extraction buffer (metaphosphoric acid, 6% p/v, 4ºC; Sigma-Aldrich; Madrid, Spain) using a microdismembrator (B-Braun mikrodismembrator-II; AG; Melsungen, Germany). The homogenate was then transferred to Eppendorf tubes (2.5 mL) and centrifuged (Jouan MR 1812 centrifuge; Saint Nazaire, France) for 4 min at 10,000 *g* and 4ºC. The supernatant was decanted into fresh Eppendorf tubes and the ascorbate content was analyzed immediately.

For cellular G6PDH activity determinations, extracts were processed in the same way, but in this case using 1.5 mL of buffer (66 m*M* $K_2HPO_4/KHPO_4$, 1 m*M* EDTA, 10 m*M* $MgCl_2$, and 14m*M* DTT; pH 7.6; Sigma-Aldrich; Madrid, Spain). Enzymatic activity was analyzed immediately after the extraction process.

### Extraction of Apoplastic Fluid

The extraction of apoplastic fluid was carried out following the protocol of Takahama and Oniki[37]. Flag leaf fragments (8–10 cm long) were weighed, washed with distilled water, and vacuum-infiltrated at –80 kPa (five periods each of 30 s) with cold (4ºC) buffer (pH 3.2) containing 66 m*M* $KH_2PO_4$ (Sigma-Aldrich; Madrid, Spain), 100 m*M* KCl (Sigma-Aldrich; Madrid, Spain), and 2.5 m*M* EDTA (Sigma-

Aldrich; Madrid, Spain). Each leaf was carefully blotted with filter paper until dry, weighed again, and then rolled and inserted into a syringe (10 × 1.5 cm). Apoplastic washing fluid was collected in Eppendorf tubes containing 100 μL of cold metaphosphoric acid (2% p/v, 4ºC; Sigma-Aldrich; Madrid, Spain) under centrifugation (6 min at 900 *g*). Subsequently, leaf fragments were collected and weighed for a third time. The amount of apoplastic fluid was obtained by weight difference. The amount of ascorbate was analyzed immediately.

For the analysis of G6PDH activity in apoplastic fluid, the infiltrations were performed following a similar protocol, but using a different buffer (66 m*M* $K_2HPO_4/KHPO_4$, 1 m*M* EDTA, 100 m*M* KCl, 10 m*M* $MgCl_2$, and 14m*M* DTT; pH 7.6; Sigma-Aldrich; Madrid, Spain). Then, the leaf fragments were collected, blotted with filter paper, inserted into a syringe (10 × 1.5 cm), and centrifuged (6 min at 900 *g*, 4ºC). The apoplastic washing fluid was collected in empty Eppendorf tubes and G6PDH activity was analyzed immediately.

Apoplastic fluid was extracted to assess its pH. Eight to 10 central flag leaf fragments (8–10 cm) were cut in half perpendicular to leaf length to increase the fluid exit surface. Infiltration was performed with ice-cold buffer (4ºC; 66 m*M* $KH_2PO_4$, 100 m*M* KCl, and 2.5 m*M* EDTA; pH 3.2; Sigma-Aldrich; Madrid, Spain), collecting the apoplastic washing fluid (<50 μL) in empty Eppendorf tubes under centrifugation (4ºC, 5 min at 900 *g*). The pH of the apoplastic washing fluid was assessed immediately with a microelectrode (6 mm Ø) fitted to a pH meter (Crison micropH 2001; Madrid, Spain) at 25ºC[44].

### G6PDH Activity Determinations

G6PDH activity was assessed following the reduction of NADP to NADPH at 340 nm under spectrophotometry (Perkin Elmer UV/VIS Lambda 2; Wellesley, MA)[22]. The extinction coefficient used for NADPH was 6.22 $mM^{-1}$ $cm^{-1}$. Absorbance was assessed at 25ºC using calibrated quartz cuvettes (1 mL) containing 50 μL of sample in buffer (66 m*M* $K_2HPO_4/KHPO_4$ [pH 7.6], 10 m*M* $MgCl_2$, 300 μ*M* NADP, and 2 m*M* G6P to start the reaction; Sigma-Aldrich; Madrid, Spain) up to a final volume of 1 mL. Enzyme activity was assessed as the increase in absorbance at 340 nm since under the above conditions, NADP continued to be transformed into NADPH. Finally, the percentage of apoplastic contamination by cytoplasmic fluid was determined as follows: [(apoplastic G6PDH activity)/(cytoplasmic G6PDH activity)] × 100.

### Ascorbate Determinations

The assessment of apoplastic and symplastic ascorbate and dehydroascorbate levels was carried out by spectrophotometry (Perkin Elmer UV/VIS Lambda 2; Wellesley, MA) following the protocol of Takahama and Oniki[37]. All assays were performed at 25ºC using calibrated quartz cuvettes (1 mL) containing 50 μL of sample in buffer (1 *M* $K_2HPO_4/KHPO_4$, pH 6.1, Sigma-Aldrich; Madrid, Spain) up to a final volume of 1 mL. The amount of ascorbate was determined by applying the Lambert-Beer law, at 265 nm, using an extinction coefficient of 14.3 $mM^{-1}cm^{-1}$:

$$A = \varepsilon\, b\, c \qquad \text{(Eq. 1)}$$

where A is absorbance at a fixed wavelength, ε is the extinction coefficient ($M^{-1}cm^{-1}$) at the above wavelength (265 nm), b is the light-beam path length through the sample (cm), and c is the sample concentration (*M*).

Two replicates were assessed for each sample: 5 μL of ascorbate-oxidase (250 U per 250 μL of buffer; Sigma-Aldrich; Madrid, Spain) was added to one of the replicates in order to elicit the oxidation of ascorbate to dehydroascorbate; 5 μL of dithiotreitol (DTT: 16 mg per 250 μL of buffer; Sigma-Aldrich;

Madrid, Spain) was added to the second replicate in order to induce the reduction of dehydroascorbate to ascorbate. After 2 min of incubation, a second absorbance reading was taken for each replicate. Ascorbate and dehydroascorbate contents were determined from the differences between the two readings. The ascorbate redox status was assessed as: [AA/(AA + DHA)] × 100.

### Mesophyll Resistance Calculations

Mesophyll resistance ($r_m$) was calculated using the SODA model[34]. This model further expands the method proposed by Chameides[3] for the estimation of ozone flux to the plasmalemma since it considers the role played by apoplastic ascorbate as the first detoxification system of the cell against ozone. The model calculates the ozone flux that enters the leaf through the stomata and that will react with apoplastic ascorbate, and it is based on current knowledge of the parameters characterizing the ozone-ascorbate reaction. Apoplastic ascorbate was determined from the total cellular ascorbate content (AA + DHA), considering that the distribution of ascorbate and dehydroascorbate in the various subcellular compartments, the rate of ascorbate transport to the apoplast, and leaf geometry and ultrastructure do, in fact, affect the distribution of ozone and ascorbate in the cell. As well as mesophyll resistance, the model also estimates the percentage of ozone reacting with apoplastic ascorbate and the ozone flux to the plasmalemma.

The model requires certain standardized input parameters (Table 1) and some changeable input data (mean hourly values):

- Total leaf ascorbate concentration (AA + DHA, m*M*)
- Apoplastic pH
- Stomatal conductance to water vapor ($g_s$, mmol $m^{-2}$ $s^{-1}$)
- Ozone concentration at canopy height (ppb)

For each group of input data, the model provides the following output parameters:

- Mesophyll resistance to the entry of ozone into the cell ($r_m$, s $m^{-1}$; Fig. 1)
- Ozone flux to the plasmalemma ($F_m$, nmol $m^{-2}$ $s^{-1}$; Fig. 2)
- Percentage of ozone reacting with apoplastic ascorbate (% $O_3$ reacted; Fig. 3)

**TABLE 1**
**Standardized Input Values Employed**

| Input | Value | Reference |
|---|---|---|
| Cell wall thickness | 0.396 µm | Experimental |
| Mesophyll cell surface area | 3.4 $m^2$ $m^{-2}$ | Experimental |
| Chloroplast volume | 0.0022 L $m^{-2}$ | Experimental |
| Cell wall tortuosity factor | 0.3 | Nobel[29] |
| $O_3$-ASC reaction rate constant | $4.8 \times 10^7$ $M^{-1}$ $s^{-1}$ | Kanofsky and Sima[20] |
| ASC:$O_3$ reaction stoichiometry | 2:1 | Van der Vliet et al.[42] |
| Temperature | 25ºC | — |

### Statistical Analysis

The main parameters were studied with regression analysis (Statistica 7.0, StatSoft, Inc., 1996).

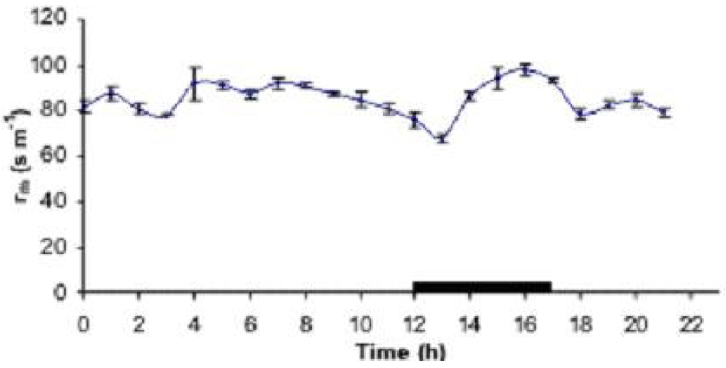

**FIGURE 1.** Mesophyll resistance ($r_m$), 24-h profile. Vertical bars indicate ± SE. Horizontal thick bar indicates the period of maximum values of ozone. Time is set from 0 to 23 h. n = 24.

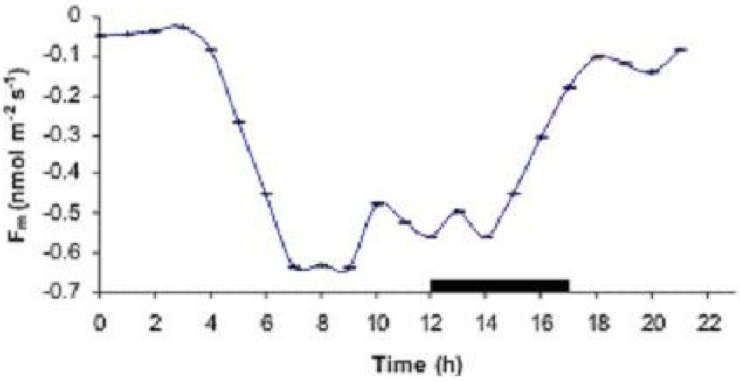

**FIGURE 2.** Ozone flux to the plasmalemma ($F_m$), 24-h profile. Vertical bars indicate ± SE. Horizontal thick bar indicates the period of maximum values of ozone. Time is set from 0 to 23 h. n = 24.

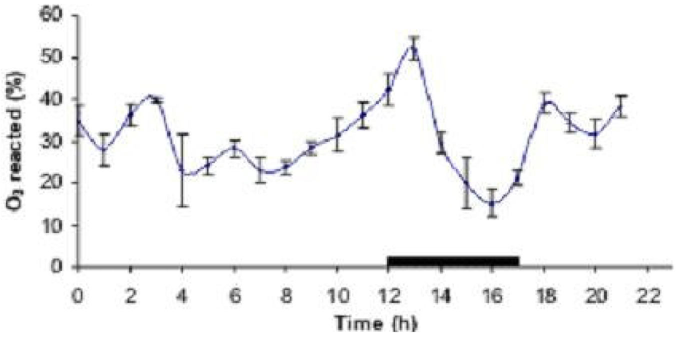

**FIGURE 3.** Ozone reacted (%), 24-h profile. Vertical bars indicate ± SE. Horizontal thick bar indicates the period of maximum values of ozone. Time is set from 0 to 23 h. n = 24.

## RESULTS

### Ambient Ozone Concentrations

The ambient ozone concentrations showed a typical profile, with maximum values seen at midday (40–50 ppb) from 12 to 17 h and minimum values at night (15–20 ppb) from 3 to 7 h.

### Stomatal Conductance

Stomatal conductance ($g_s$) was below 50 mmol $m^{-2}$ $s^{-1}$ at night, increasing in the morning, with a maximum at 9 h (333 mmol $m^{-2}$ $s^{-1}$), and decreasing at midday to 150 mmol $m^{-2}$ $s^{-1}$, with a partial stomatal closure due to vapor pressure deficit (VPD) values greater than 2.5 kPa.

### Ozone Stomatal Fluxes

Ozone stomatal fluxes (OSF) were calculated according to:

$$\text{OSF (nmol m}^{-2}\text{ s}^{-1}) = g_s\,(O_3)[O3] \qquad \text{(Eq. 2)}$$

where $g_s$ ($O_3$) is the stomatal conductance to ozone (mol $m^{-2}$ $s^{-1}$) and [$O_3$] is the ambient ozone concentration (nmol $mol^{-1}$). Thus,

$$g_s\,(O_3) = g_s\,D_r \qquad \text{(Eq. 3)}$$

where $D_r = D_{O3}/D_{H2O} = 0.613$ is the molecular diffusivity rate of ozone to water vapor[1].

### Biochemical Analyses

The biochemical analyses revealed ascorbate mean levels of about 12 m*M* and dehydroascorbate levels of 0.3–1.5 m*M* in the symplasm. In the apoplast, the reduced ascorbate level was about 0.2–1 m*M* (Fig. 4), and that of dehydroascorbate was 0.11–0.77 m*M*, the latter representing 35–77% of total ascorbate in the apoplast (considering hourly minimum and maximum values, respectively). The experimental pH values lay between 5.63 and 6.01.

### Application of SODA Model

The SODA model provided the 24-h profiles for mesophyll resistance ($r_m$, s $m^{-1}$) (Fig. 1), ozone flux to the plasmalemma ($F_m$, nmol $m^{-2}$ $s^{-1}$) (Fig. 2), and the percentage of ozone reacting with apoplastic ascorbate (% $O_3$ reacted) (Fig. 3).

## DISCUSSION

### Biochemical Analysis

Fig. 4 shows a significant peak for ascorbate in the apoplast at 13 h that could be a response to the high ozone fluxes recorded between 9 and 11 h due to high stomatal conductances and relatively high ozone

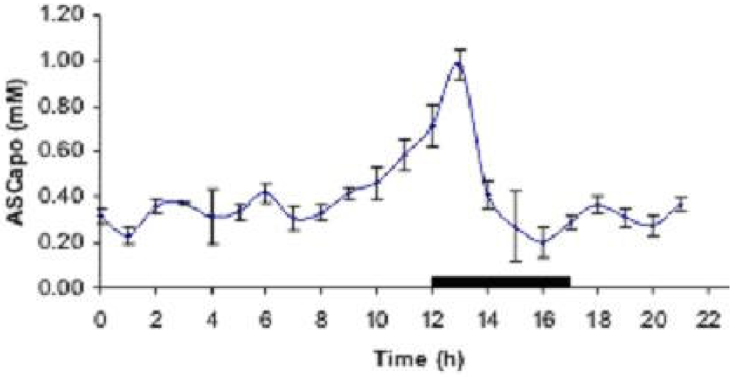

**FIGURE 4.** Reduced apoplastic ascorbic acid ($ASC_{apo}$), 24-h profile. Vertical bars indicate ± SE. Horizontal thick bar indicates the period of maximum values of ozone. Time is set from 0 to 23 h. n = 24.

concentrations. This would be in agreement with the increasing apoplastic ascorbate levels induced by exposure to ozone described for other species[30,35]. It is also in accordance with the results of Yoshida and collaborators[43], who found that exposure to ozone in *Arabidopsis* increased the amount of cytosolic dehydroascorbate reductase, which is responsible for the reduction of dehydroascorbate to ascorbate.

The highest pH was recorded between 12–14 h, coinciding with the highest apoplastic ascorbate levels. High values of ascorbate should decrease the pH, but in our case, the opposite was seen. Some authors have reported that the presence of ozone modifies the pH conditions and the delicate electrical charge equilibrium that controls the active transport of substances through the membrane[18], and this could be the reason of the increasing of the pH values.

### Application of SODA Model

Figs. 1, 2, and 3, corresponding to the 24-h profiles for mesophyll resistance ($r_m$, s $m^{-1}$), ozone flux to the plasmalemma ($F_m$, nmol $m^{-2}$ $s^{-1}$), and the percentage of ozone reacting with apoplastic ascorbate (% $O_3$ reacted), respectively, show the minimum mesophyll resistance ($r_m$), a relatively low ozone flux to the plasmalemma ($F_m$) and the maximum $O_3$ reacted at midday (13 h), all coinciding with maximum apoplastic ascorbate. The ascorbate profile is consistent with the results of Chen and Gallie[5], i.e., almost constant during the early morning, with maximum values at midday, decreasing in the afternoon, and recovering by night, probably indicating that ascorbate levels are diurnally regulated. This suggests that maximum $O_3$ reacts with maximum apoplastic ascorbate, as their maximum values coincide in time[2,34].

### Regression Analysis

- **Apoplastic ascorbate (AA) vs. $O_3$:** A hemiparabolic-type relationship ($R^2$ = 0.59; $p < 0.05$) was found, i.e., ascorbate seemed to increase as $O_3$ did. When $O_3$ > 30 ppb was correlated with AA levels, a better exponential regression was obtained ($R^2$ = 0.66; $p < 0.05$; Fig. 5), which means that ascorbate increases, especially when high $O_3$ concentrations occur. In other words, ascorbate seems to be directly correlated with the ozone concentration[2]. Concerning the increase in ascorbate at midday, it has already been reported that a diurnal regulation of ascorbate levels occurs that might be related to maximum daylight intensities and that may be correlated with photosynthetic activity[5]. Despite this, it is well known that maximum ozone concentrations coincide with maximum ascorbate values and, because of this, an enhanced tolerance to ozone is found[6].

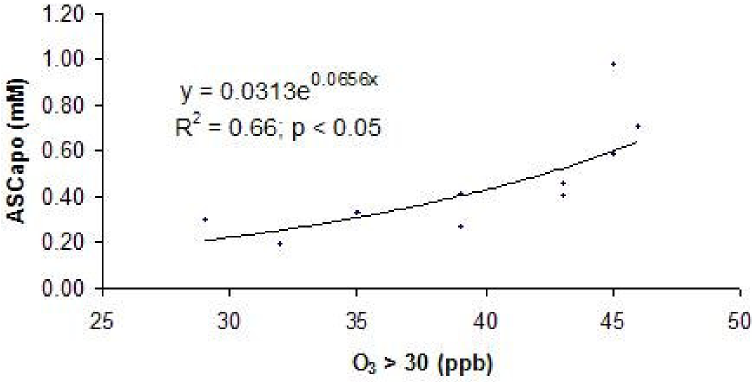

**FIGURE 5.** Regression for reduced apoplastic ascorbic acid (ASCapo) and ozone concentrations higher than 30 ppb ($O_3$ > 30 ppb). n = 10.

- **% $O_3$ reacted vs. $O_3$:** The same pattern as in the previous section was observed, i.e., a parabolic relationship between % $O_3$ reacted and $O_3$ ($R^2 = 0.35$; $p < 0.05$); when $O_3 > 30$ ppb was correlated with the % of $O_3$ reacted, a better exponential regression was obtained ($R^2 = 0.66$; $p < 0.05$), meaning that the % of $O_3$ reacted increases considerably when high $O_3$ concentrations occur (the % $O_3$ reacted is directly correlated to ozone), as would be expected.
- **% $O_3$ reacted vs. apoplastic ascorbate (AA):** Based on both previous regressions, the % $O_3$ reacted is directly related to ascorbate, i.e., the greater the increase in ascorbate, the greater the increase in the % of $O_3$ reacted with ascorbate[2,34]. These results are in agreement with those of Chen and Gallie[6], who found that after chronic or acute exposure to ozone, a marked reduction in ascorbate levels is seen, suggesting that ascorbate is used in the detoxification of this gas and, hence, the highest amount of ozone reacted occurs at maximum ascorbate levels. In our study, the plants were not subjected to chronic exposure to especially high ozone concentrations (14 days, ambient concentrations vs. 30 days at 100 ppb in the Chen and Gallie study[6]), and ozone exposure at midday cannot be considered as truly acute (around 45–50 ppb vs. 200 ppb for 2 h in the Chen and Gallie study[6]). Nevertheless, ascorbate was markedly reduced after midday (see Fig. 4). Two patterns were observed: one for the night hours (N, night) and one for the day hours (D, day). Both are very similar, and with a high amount of variance explained ($R^2$_N = 0.97; $p < 0.001$; $R^2$_D = 0.98; $p < 0.001$; Fig. 6). The main difference is the steeper slope of the night-time relationship ($m_N$ = 76.989 vs. $m_D$ = 46.12 for the day-time relationship), indicating a faster response by ascorbate to react with $O_3$ at night, while the capacity to react with $O_3$ during the daylight hours is greater (up to 52%), probably because of the large pool of ascorbate.
- **Ozone flux to the plasmalemma ($F_m$) vs. stomatal conductance ($g_s$):** This relationship is linear and direct; i.e., the greater the increase in stomatal conductance, the greater the increase in ozone flux to the mesophyll ($R^2 = 0.72$; $p < 0.05$), as also expected.
- **Mesophyll resistance ($r_m$) vs. ozone ($O_3$ > 30 ppb):** No clear regression for the overall ozone data was found, but a much clearer relationship was seen when $O_3 > 30$ ppb, with an inverse linear regression ($R^2 = 0.71$; $p < 0.01$), indicating that as $O_3$ increases, the $\underline{r_m}$ decreases. This is because the highest $O_3$ concentrations are found at midday, when $\underline{r_m}$ is minimum, probably because of the % $O_3$ reacting with apoplastic ascorbate.
- **Mesophyll resistance ($r_m$) vs. apoplastic ascorbate (AA):** A very marked linear and inverse regression was found ($R^2$ = 0.97–0.98; $p < 0.001$; Fig. 7). Again, this is because ascorbate is maximum when mesophyll resistance is minimum and vice versa; as ascorbate increases, mesophyll resistance decreases. A dual linear regression was observed, (as in the % $O_3$ reacted vs. ascorbate): one for the night-time hours (N, night) and the other for the day-time hours (D, day). As before, a steeper slope was seen for the night time hours ($m_N$ = –62.89, vs. $m_D$ = –38.072), indicating a faster

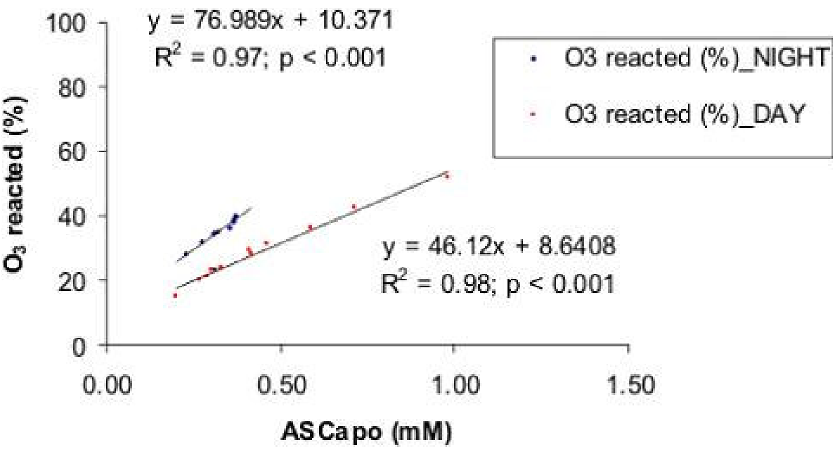

**FIGURE 6.** Regression for ozone reacted ($O_3$ reacted) and reduced apoplastic ascorbic acid ($ASC_{apo}$) for night-time (upper line, $O_3$ reacted (%)_NIGHT; n = 10) and day-time hours (lower line, $O_3$ reacted (%)_DAY; n = 14).

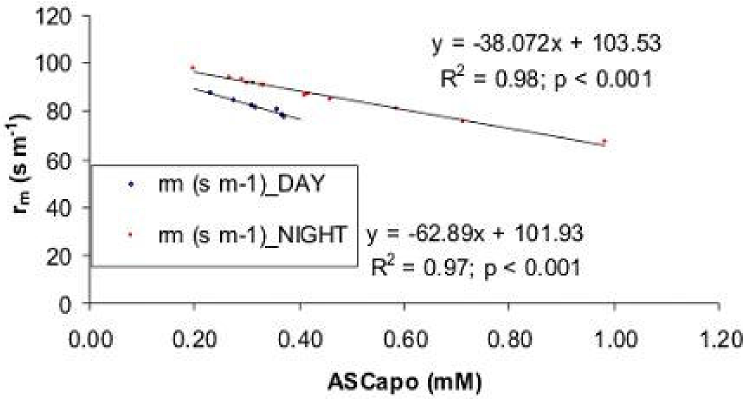

**FIGURE 7.** Regression for mesophyll resistance ($r_m$) and reduced apoplastic ascorbic acid ($ASC_{apo}$) for night-time (upper line, $O_3$ reacted (%)_NIGHT; n = 10) and day-time hours (lower line, $O_3$ reacted (%)_DAY; n = 14).

decrease in mesophyll resistance at night for small increases in ascorbate, while higher ascorbate levels in the day are associated with lower decreases in $r_m$.

- **Ozone flux to the plasmalemma ($F_m$) vs. apoplastic ascorbate:** On considering all the data together, a weak logarithmic regression was obtained ($R^2 = 0.26$; *p n.s.*), and it seems that $F_m$ tended to increase with ascorbate. However, this increase in $F_m$ is attenuated, probably due to the reaction of ascorbate with $O_3$. Nevertheless, if only high $F_m$ values are considered ($F_m > 0.47$ nmol $m^{-2}$ $s^{-1}$), corresponding to day-time values, an inverse linear regression is found ($R^2 = 0.71$; $p < 0.05$; Fig. 8). This suggests that when $F_m$ increases sufficiently, ascorbate reacts with $O_3$, decreasing the $F_m$. It is noteworthy that maximum $F_m$ values were observed between 7 and 9 h, when ascorbate is still low and not very different from night-time values. As a response to such high $F_m$ values, ascorbate tended to increase during the morning and $F_m$ was reduced to intermediate values (see Figs. 2 and 4).
- **Ozone flux to the plasmalemma ($F_m$) vs. % $O_3$ reacted:** Exactly the same pattern was obtained as in the previous case; this is fairly logical, bearing in mind the strong direct linear regression between the percent of $O_3$ reacted and ascorbate. In this case, the logarithmic regression was even weaker ($R^2 = 0.17$; *p n.s.*) and the linear one was even stronger ($R^2 = 0.83$; $p < 0.05$).

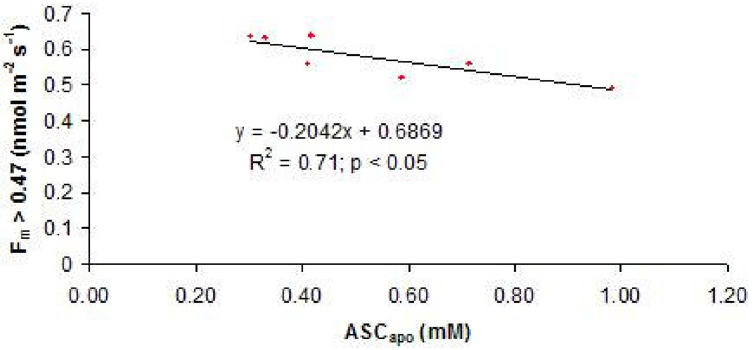

**FIGURE 8.** Regression for ozone flux to the plasmalemma ($F_m$) higher than 0.47 nmol $m^{-2}$ $s^{-1}$ and reduced apoplastic ascorbic acid ($ASC_{apo}$). n = 7.

A multiple regression was obtained for apoplastic ascorbate as the dependent variable and $r_m$, $F_m$, and the % $O_3$ reacted as independent variables. The result was a very strong correlation ($R^2$ = 0.99; $p$ = 0.00046), i.e., those three parameters were able to explain almost 100% of the variance in apoplastic ascorbate.

### CONCLUSIONS

The computer model used in this experiment, SODA, has been shown to afford accurate results and is able to simulate a realistic ozone detoxification in the apoplast by ascorbate for a Mediterranean wheat cultivar (*Triticum durum* Desf. Cv. Camacho). Degrees of ozone detoxifications up to 52% were found at midday, when maximum ozone concentrations and maximum apoplastic ascorbate were present. Apoplastic ascorbate highest levels seem to coincide with the highest ambient ozone concentrations and, hence, with the percentage of ozone reacted, especially during the day, when a higher detoxification capacity was observed. Mesophyll resistance was minimum at this time and the ozone flux to the plasmalemma was reduced because of ozone depletion by apoplastic ascorbate. The regression analysis of apoplastic ascorbate vs. ozone showed a good correlation, and as a consequence of that, the regression analysis of apoplastic ascorbate vs. percent of ozone detoxification was also very good, because apoplastic ascorbate and ozone maximum values coincide in time and apoplastic ascorbate has been demonstrated to be an excellent antioxidant against ozone. To amplify that matter, it would have been desirable to compare the daily cycle of apoplastic ascorbate of the plants studied with the ascorbate cycle for plants not subjected to ozone exposure, thus enabling speculation about which factors would be responsible for the increases and decreases in ascorbate levels, since ozone is not in fact the only factor that affects the ascorbate cycle. Nevertheless, this was not the aim of our study; instead, we wished to check the effectiveness and accuracy of the SODA model applied to a Mediterranean wheat cultivar. The SODA model results indicated that apoplastic ascorbate reacts with ozone, ascorbate being one of the main antioxidants that protect plants, as it has been already and largely cited in the scientific literature.

### ACKNOWLEDGMENTS

Thanks are given to Dr. Agueda González Rodriguez (IMIDRA) for her intermediation with her research institute in order to be able to make the experimental measurements at El Encin.
